# Selective Production of 9*R*-Hydroxy-10*E*,12*Z*,15*Z*-Octadecatrienoic Acid from α-Linolenic Acid in Perilla Seed Oil Hydrolyzate by a Lipoxygenase from *Nostoc* Sp. SAG 25.82

**DOI:** 10.1371/journal.pone.0137785

**Published:** 2015-09-17

**Authors:** Kyoung-Rok Kim, Jung-Ung An, Seon-Hwa Lee, Deok-Kun Oh

**Affiliations:** Department of Bioscience and Biotechnology, Konkuk University, Seoul, Republic of Korea; USDA-ARS, UNITED STATES

## Abstract

Hydroxy fatty acids (HFAs) derived from omega-3 polyunsaturated fatty acids have been known as versatile bioactive molecules. However, its practical production from omega-3 or omega-3 rich oil has not been well established. In the present study, the stereo-selective enzymatic production of 9*R*-hydroxy-10*E*,12*Z*,15*Z*-octadecatrienoic acid (9*R*-HOTE) from α-linolenic acid (ALA) in perilla seed oil (PO) hydrolyzate was achieved using purified recombinant 9*R*-lipoxygenase (9*R*-LOX) from *Nostoc* sp. SAG 25.82. The specific activity of the enzyme followed the order linoleic acid (LA) > ALA > γ-linolenic acid (GLA). A total of 75% fatty acids (ALA and LA) were used as a substrate for 9*R*-LOX from commercial PO by hydrolysis of *Candida rugosa* lipase. The optimal reaction conditions for the production of 9*R*-HOTE from ALA using 9*R*-LOX were pH 8.5, 15°C, 5% (v/v) acetone, 0.2% (w/v) Tween 80, 40 g/L ALA, and 1 g/L enzyme. Under these conditions, 9*R*-LOX produced 37.6 g/L 9*R*-HOTE from 40 g/L ALA for 1 h, with a conversion yield of 94% and a productivity of 37.6 g/L/h; and the enzyme produced 34 g/L 9*R*-HOTE from 40 g/L ALA in PO hydrolyzate for 1 h, with a conversion yields of 85% and a productivity of 34 g/L/h. The enzyme also converted 9*R*-hydroxy-10*E*,12*Z*-octadecadienoic acid (9*R*-HODE) from 40 g/L LA for 1.0 h, with a conversion yield of 95% and a productivity of 38.4 g/L. This is the highest productivity of HFA from both ALA and ALA-rich vegetable oil using LOX ever reported. Therefore, our result suggests an efficient method for the production of 9*R*-HFAs from LA and ALA in vegetable oil using recombinant LOX in biotechnology.

## Introduction

Lipoxygenase (LOX) catalyzes the regio- and stereo-selective hydroperoxidation of free polyunsaturated fatty acids (PUFAs) possessing one or more (1*Z*,4*Z*)-pentadienes to form hydroperoxy fatty acids (HPFAs) that reduced to hydroxy fatty acids (HFAs) [1]. These hydroxyperoxy- and hydroxy-fatty acids act as precursors for the biosynthesis of signaling molecules by further enzymatic cascade reactions and have a variety of functions [2, 3]. Extensive research has been performed using plant and animal LOX systems to study the physiological functions [4]. However, recent reports have described the different substrate specificity and stereo-selectivity of bacterial and fungal LOXs for omega-3 PUFAs compared to those of plant and animal LOXs [5–7].

To date, LOX has mainly been applied to the production of HPFAs, which are utilized to produce green note volatiles combined with hydroperoxy lyase [8]. Due to its highly reactive hydroperoxy forming activity, LOX is also used to modify the food polymers including wheat proteins [9, 10]. Application of LOX for pulp processing from lignocellulogic agro-materials has shown a variety of peroxy products [11]. Interestingly, new emerging applications of LOX include the production of HFAs, because they are widely used as starting materials for the synthesis of a variety of polymers and as additives in the manufacturing of lubricants, emulsifiers, and stabilizers in the chemical, food, cosmetic, and pharmaceutical industries, owing to their higher reactivity, solvent miscibility, and stability compared to non-hydroxylated fatty acids [12, 13]. HFAs are also notable intermediates for bio-grade flavor lactone production [14]. Therefore, the biotechnological production of HFAs from PUFAs is valuable.

α-Linolenic acid (ALA; 18:3*n*-3) is an essential fatty acid that requires dietary uptake as it is not synthesized by the human body due to the absence of endogenous desaturases such as Δ5- and Δ6-desaturases. Perilla (*Perilla frutescens*) seed oil (PO) is known to contain approximately 60% ALA [15, 16]. Therefore, PO is one of the most important and rich sources of ALA in East Asian countries, including Korea, China, and Japan, and is widely used as a vegetable oil in traditional foods. Increasing ALA uptake shows remarkable pharmacological effects in the improvement of inflammatory and immune disorders, as well as preventing in cardio-vascular disorders [17]. Furthermore, LOX-derived bioactive HFAs from ALA, including 9-hydroxy-10*E*,12*Z*,15*Z*- or 13-hydroxy-9*Z*,11*E*,15*Z*-octadecatrienoic acid (9- or 13-HOTE), have been known as antifungal [18], anti-inflammatory [19], and signal modulation compounds [20]. Although these compounds have been isolated and identified from a wide range of taxonomic origins [18, 19, 21, 22], their specific production, especially using omega-3 rich oil, is scarce. Recently, 9*R*-LOX from *Nostoc* sp. showed a wide range of substrate specificity for PUFAs that form HPFAs [6]. Therefore, *Nostoc* 9*R*-LOX can be applied to produce HFA from ALA in omega-3 rich vegetable oil.

In the present study, the stereo-selective biocatalyst 9*R*-LOX from *Nostoc* sp. was expressed in *Escherichia coli* and purified. The purified enzyme was applied to the production of 9*R*-HOTE. The reaction conditions, including pH, temperature, organic solvent, detergent, and the concentrations of enzyme and substrate, were optimized. Under the optimized conditions, the stereo-specific production of 9*R*-HOTE was attempted using ALA and ALA-rich PO.

## Materials and Methods

### Chemicals and reagents

High-quality (>99%) ALA and linoleic acid (LA; 18:2*n*-6) were purchased from Nu-Check Prep (Elysian, MN) and Sigma (St. Louis, MO, USA), respectively. 9-Hydroperoxy-trioctadecatrienoic acid (9-HPOTE) and 9*S*-HOTE standards were purchased from Cayman Chemicals (Ann Arbor, MI). 9*R*-HOTE standard was prepared by the previously reported procedure [23]. Solvents, including 2-propanol, acetonitrile, *n*-hexane, and methanol, were high-performance liquid chromatography (HPLC) grade and ethanol and ethyl acetate for extraction were reagent grade. These solvents were supplied from Duksan Chemical (Ansan, Korea). Commercial PO was obtained from a local market. Luria-Bertani (LB) medium was purchased from Fermentas (Vilnius, Lithuania). Ampicillin, boric acid, and isopropyl-β-d-thiogalactopyranoside (IPTG) were purchased from USB (Cleveland, OH, USA). Lipases from recombinant *Aspergillus oryzae* (AOL), *Candida rugosa* (CRL), *Pseudomonas cepacia* (PCL), *Pseudomonas fluorescense* (PFL), *Rhizomucor meihei* (RML), and *Thermomyces lanoginosus* (TLL) were obtained from Sigma.

### Gene expression

The constructed plasmid harboring *Nostoc* sp. 9*R*-lipoxygenase gene (*lox*) in pET-15b (9*R*-*lox*/pET-15b) was used for expression in *E*. *coli* [23]. This plasmid was transformed into *E*. *coli* ER2566 cells by electroporation and selected on LB agar containing 50 μg/ml ampicillin. A single colony of *E*. *coli* cells containing the 9*R*-*lox*/pET-15b plasmid were inoculated into 3 ml of liquid LB medium and cultivated at 37°C with shaking at 220 rpm overnight. This culture was transferred into a 2,000 ml flask containing 500 ml of LB medium and 50 μg/ml ampicillin at 37°C with shaking at 200 rpm. When the optical density of the culture reached 0.6 at 600 nm, expression of the 9*R*-*lox* gene was induced by adding isopropyl-β-D-thiogalactopyranoside with a final concentration of 0.1 mM, and the culture was further incubated for 16 h at 16°C with shaking at 150 rpm.

### Enzyme preparation

The cultured cells were harvested by centrifugation at 6000×g for 20 min at 4°C and washed twice with 0.85% (w/v) NaCl. The cells were disrupted by sonication on ice for 2 min in 50 mM Tris-HCl buffer (pH 8.0) containing 300 mM NaCl and 1 mg/ml lysozyme. The unbroken cells and debris were removed by centrifugation at 13000×g for 20 min at 4°C, and the obtained supernatant was used as a crude extract. Purification was carried out using a fast protein liquid chromatography system (Bio-Rad) in a cold room at 4°C. The crude extract was applied to a His-Trap affinity chromatography column (GE Healthcare, Piscataway, NJ, USA) equilibrated with 50 mM Tris-HCl buffer (pH 8.0). The bound enzyme was then eluted at 4°C with the same buffer containing 250 mM imidazole at a flow rate of 1 ml/min. The active fractions were collected and dialyzed at 4°C for 16 h against 50 mM Tris-HCl buffer (pH 8.5). The resulting solution was used as the purified enzyme.

### Determination of LOX activity and enzyme assay

LOX activity was determined by measuring conjugated diene formation at 234 nm using a Beckman Coulter DU-700 spectrophotometer (Brea, CA, USA). An extinction coefficient of 25,000 M^−1^ cm^−1^ was used to calculate the activity of the enzyme for 9-HPODE or 9-HPOTE [24, 25]. Unless otherwise stated, the reaction was performed in 0.45 ml of 50 mM Tris-HCl buffer (pH 8.5) containing 50 μL substrate (10 mM ALA or LA), 0.05% (w/v) Tween 80, and 0.2−10 μg enzyme at 25°C for 5 min. The range was adjusted and monitored to give a slope within A_234_ < 1.0. One unit (U) of LOX activity is defined as the amount of enzyme catalyzing the formation of 1 μmol of product per min. The rate of the reaction was compared to that without the addition of enzyme as a control.

### Effects of pH and temperature

To investigate the effects of pH and temperature on 9*R*-LOX activity for the production of 9*R*-HOTE from ALA, the pH values were varied from 7.0 to 10.0 using 50 mM Tris-HCl buffer (pH 7.0–9.0) and 0.2 M sodium borate buffer (pH 9.0–10.0) at a constant temperature of 25°C, while the temperatures were varied from 10 to 50°C at a constant pH of 8.0. The effect of temperature on enzyme stability was monitored for 4 h by incubating the enzyme solution at different temperatures from 5 to 50°C in 50 mM Tris-HCl buffer (pH 8.5). Samples were withdrawn at time intervals and then assayed in 50 mM Tris-HCl buffer (pH 8.5) containing 0.25 mM ALA and 0.05 U ml^−1^ enzyme at 15°C for 5 min. The half-life of the enzyme was calculated using Sigma Plot 10.0 software (Systat Software, San Jose, CA, USA).

### Effects of solvents and detergents

Several organic solvents at concentrations of 4% and 6% (v/v), including methanol, ethanol, butanol, isopropanol, heptanol, octanol, acetone, ethyl acetate, dichloromethane, hexane, and dimethyl sulfoxide (DMSO) were tested for selecting the optimum solvent. The effect of solvent concentration on LOX activity was evaluated by varying the concentration of acetone from 1% to 10% (v/v). The reactions were performed in 50 mM Tris-HCl buffer (pH 8.5) containing 0.25 mM ALA, 0.1 U/ml enzyme, and acetone at 15°C for 10 min. Several detergents, including Tween 20, Tween 40, Tween 80, Span 20, Span 60, Span 80, Triton-X 100, Brij 58, and sodium dodecyl sulfate (SDS), were tested to find the suitable detergent for the maximum production of 9*R*-HOTE from ALA. Tween 80 concentration was varied from 0.005% to 1% (w/v) to determine the optimum concentration of the detergent. The reactions were performed in 50 mM Tris-HCl buffer (pH 8.5) containing 0.25 mM ALA, 0.1 U/ml enzyme, 5% (v/v) acetone, and Tween 80 at 15°C for 10 min.

### Determination of kinetic parameters

Determination of all kinetic measurements was obtained by monitoring the time-dependent consumption of O_2_ using a Clark-type electrode (oxygen probe, YSI Model 5331, Yellow Springs, OH, USA) with a high sensitivity membrane and oxygen monitor (YSI Model 5300A) in a thermostatic oxygen cell with a cell volume of 1.5 ml at 25°C [5, 24, 26]. The cell was filled with air-saturated 50 mM Tris-HCl buffer (pH 8.5) containing substrate (0.1 mM), and was then allowed to equilibrate for approximately 5 min under a controlled O_2_/N_2_ atmosphere. The reactions were initiated by the addition of 1−5 μL concentrated enzyme, corresponding to 0.1−2 U/mg specific activity, via a gastight syringe. Nitrogen was used to adjust to different O_2_ concentrations. Enzyme activity was determined from the initial rate of oxygen uptake and consumption, as reported previously [24, 26]. LA, ALA, and γ-linolenic acid (GLA) at 100 μM were used as substrates to determine kinetic parameters. The kinetic parameters were calculated by nonlinear regression fitting of the experimental points to the Michaelis–Menten equation.

### Effects of enzyme and substrate concentrations on 9*R*-HOTE production

The optimal concentrations of enzyme and substrate for the increased production of 9*R*-HOTE from ALA by recombinant 9*R*-LOX were determined by varying the enzyme concentration from 0.05 to 2 mg/ml at a constant ALA concentration of 10 g/L and varying the substrate concentration from 5 to 50 g/L at a constant enzyme concentration of 0.5 mg/ ml. The reactions were performed in 50 mM Tris-HCl buffer (pH 8.5) containing different concentration of ALA and enzyme at 15°C for 20 min.

### Production of 9*R*-HOTE and 9*R*-HODE from ALA and LA

The production of 9*R*-HOTE from ALA by recombinant 9*R*-LOX was performed in 50 mM Tris-HCl buffer (pH 8.5) containing 40 g/L ALA, 5% (v/v) acetone, and 0.2% (w/v) Tween 80 at 15°C for 80 min. 9*R*-HODE production from LA was carried out under the same conditions as the production of 9*R*-HOTE from ALA.

### Production of 9*R*-HOTE and 9*R*-HODE from PO hydrolyzate

PO with a high content of ALA was hydrolyzed using AOL, CRL, PCL, PFL, RML, and TLL. The reaction was carried out in 9 ml of 50 mM Tris-HCl buffer (pH 7.5) containing 10 g/L PO, 1 g/L CRL (700 U/mg), and 0.02% (w/v) Tween 80 at 37°C with shaking at 250 rpm for 2 h. The reaction was stopped by boiling the reaction mixture at 100°C for 50 min. The sample was kept at −20°C for analyzing the concentrations of fatty acids. The samples were extracted twice using ethyl acetate and dried under reduced pressure. The dried samples were collected and used for the production of 9*R*-HOTE. The time course reactions for the production of 9*R*-HOTE from ALA by 9*R*-LOX were carried out in a 500 ml baffled flask in 80 ml of 50 mM Tris-HCl buffer (pH 8.5) consisting of PO hydrolyzate, which contained 40 g/L ALA, 10 g/L LA, 15.3 g/L of other fatty acids, 5% (v/v) acetone, 0.2% (v/v) Tween 80, and 1 g/L purified 9*R*-LOX at 15°C for 100 min, with agitation at 250 rpm and oxygen supplementation for efficient oxygenation. After the reactions, 9*R*-HOTE and 9*R*-HODE were obtained from 9*R*-HPODE and 9*R*-HPOTE by the addition of 8 ml of 1 M NaBH_4_, and an equal reaction volume of ethyl acetate. The ethyl acetate in the reaction mixture was removed using a rotary evaporator under reduced pressure, and the concentrations of 9*R*-HODE and 9*R*-HOTE in the reaction solutions were determined.

### Preparation of 9*R*-HOTE and 9*R*-HODE

9*R*-HOTE and 9*R*-HODE standards were prepared by a preparative HPLC after solvent extraction, as described previously [23]. The purified products were identified by chiral phase HPLC and liquid chromatography-mass spectrometry (LC-MS).

### Fatty acid analysis of PO hydrolyzate

The composition of total fatty acids from PO hydrolyzate was determined by fatty acid methyl ester (FAME) analysis, as described previously [27]. Briefly, 0.1 g of PO was mixed with 2 ml of 0.5M NaOCH_3_ and 0.05 ml of tridecanoic acid (C13:0, 0.5 mg/ml in methanol) as an internal standard in a screw-cap Pyrex tube (16×150 mm). The mixture was incubated at 55°C in a water bath for 1 h. The reactant was cooled and then 2 ml of saturated NaOCH_3_ solution and 3 ml of hexane were added in the same reaction tube for extraction. The hexane layer containing the FAME was taken into a gas chromatography (GC) vial. The vial was capped and placed at −20°C until GC analysis. A GC-flame ionization detector (FID) (Agilent 6890N) equipped with a DB-23 column (0.25 mm×30 m, 0.25 μm, J&W Scientific, Santa Clara, CA) was used for analysis. The chromatographic conditions were as follows: injector temperature, 250°C; oven temperature, started at 80°C, increased to 180°C at 25°C min^−1^, increased to 250°C at 4°C/min, and held for 5 min; column temperature, maintained at 160°C for 1 min, and then raised to 250°C at a rate of 4°C/min. Identification of compounds was confirmed by comparison with authenticated reference standards for PO (GLC-16, Nu-chek Prep). The concentrations of fatty acids were determined using the peak of the internal standard as a reference value and the standard curves of authentic compounds.

### Analysis for Substrates and Products of 9*R*-LOX

Substrates, ALA and LA were analyzed by non-specific measurement at 202 nm, while *9R* **-**LOX products, including 9-HPOTE, 9-HOTE, 9-HPODE, 9-HODE, 13-HOTE, and 13-HODE, were analyzed by specific measurement at 234 nm using an reversed-phase HPLC system (Agilent 1260, Palo Alto, CA) equipped with a UV detector and a Nucleosil C_18_ column (150 × 2.1 mm, 5 μm particle size; Phenomenex, Torrance, CA) with a guard cartridge. The column was run and eluted with a gradient of solvent A (acetonitrile/water/acetic acid; 50:50:0.1, v/v/v) and solvent B (acetonitrile/acetic acid; 100:0.1, v/v) as described [6, 23]. The chiral phase HPLC was run with a Chiralcel OD-H column (150 × 2.1 mm, 5 μm particle size; Daicel, Tokyo, Japan) and a solvent system of *n*-hexane/2-propanol/acetic acid (100:5:0.1, v/v/v) [6]. The amounts of HFAs using 9*R*-LOX were determined using linear calibration curves and correlating the peak areas to the concentrations of standards.

Liquid chromatography-mass spectrometry/mass spectrometry (LC-MS/MS) analysis of HFAs was carried out using a ThermoFinnigan LCQ Deca XP plus ion trap mass spectrometer (Thermo Scientific, Pittsburg, PA) with a Synergi fusion-RP 80Å column (100 × 30mm, 4 μm particle size; Phenomenex**)** using an electrospray ionization (ESI) interface. Two hundred microliters obtained from the HPLC fraction was concentrated under N_2_ stream to complete dryness and dissolved in 20 μl ethanol containing 0.1% acetic acid. The flow rate was 250 μl min^−1^ and a gradient of 0.1% formic acid in acetonitrile was used. The operation parameters were as follows: capillary temperature, 275°C; ion source voltage, 5 kV; nebulizer gas, 40 psi nitrogen; capillary voltage, 46 V in positive ionization mode and 15 V in negative ionization mode; average scan time, 0.01 min; average time to change polarity, 0.02 min; and collision energy (CE), approximately 35% abundance of the precursor ion.

### Statistical Analysis

The means and standard errors for all experiments, including determination of the concentrations of PUFAs and HFAs, evaluation of reaction conditions, and HPLC analysis, were calculated from triplicates. One-way analysis of variance (ANOVA) was carried out using Tukey’s method with a significance level of p < 0.05 using SigmaPlot 10.0 (Systat Software, Chicago, IL). Data were reported as mean ± standard deviation (SD) for triplicate treatments.

## Results

### Expression and purification of 9*R*-LOX from *Nostoc* sp.

**Fig 1:**
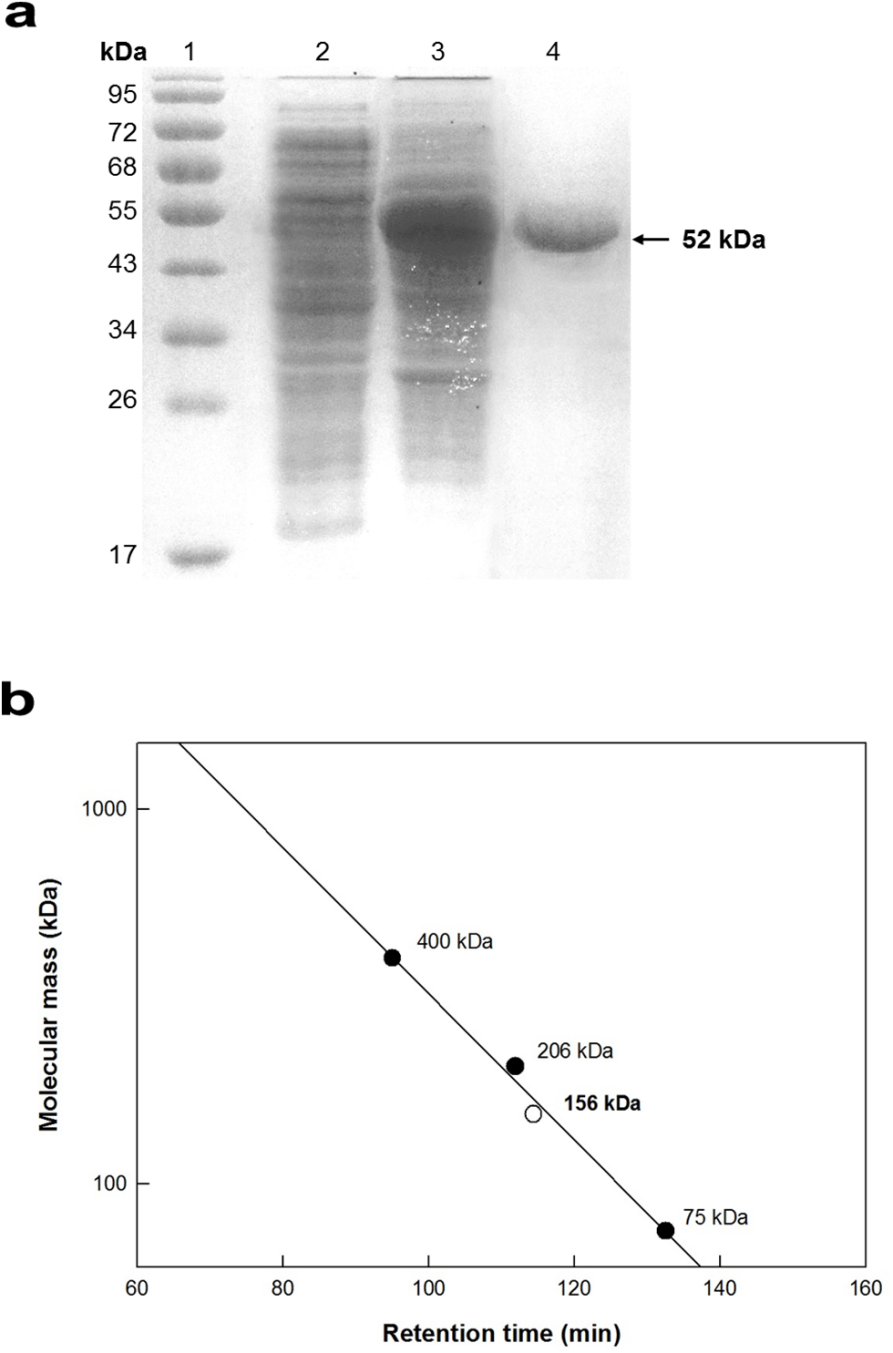
SDS-PAGE and gel-filtration chromatography of recombinant *Nostoc* sp. 9*R*-LOX. **a** SDS-PAGE analysis of each purification step. Lanes: 1, prestained marker protein (95, 72, 68, 55, 43, 34, 26 and 17 kDa); 2, control strain containing plasmid pET-15b without *lox* gene; 3, crude extract; 4, purified enzyme. **b** Gel-filtration chromatography. Ferritin (400 kDa), catalase (206 kDa), and conalbumin (75 kDa) (*filled circle*); and 9*R*-LOX (*empty circle*).

The recombinant 9*R*-LOX from *Nostoc* sp. SAG 25.82 was purified as a soluble protein from crude extract by His-Trap affinity chromatography. The molecular mass of the purified enzyme analyzed by SDS-PAGE was approximately 52 kDa (Fig 1A), which was consistent with the calculated value of 52.3 kDa from the 456 amino acid residues and 6 histidine residues, as determined using the Compute pI/Mw tool (web.expasy.org/compute_pi/). Based on the molecular masses of the reference proteins, the native enzyme may exist as a trimer with a molecular mass of 156 kDa, as determined by gel filtration chromatography (Fig 1B).

### Identification and analysis of products obtained from ALA and LA by 9*R*-LOX from *Nostoc* sp.

**Fig 2:**
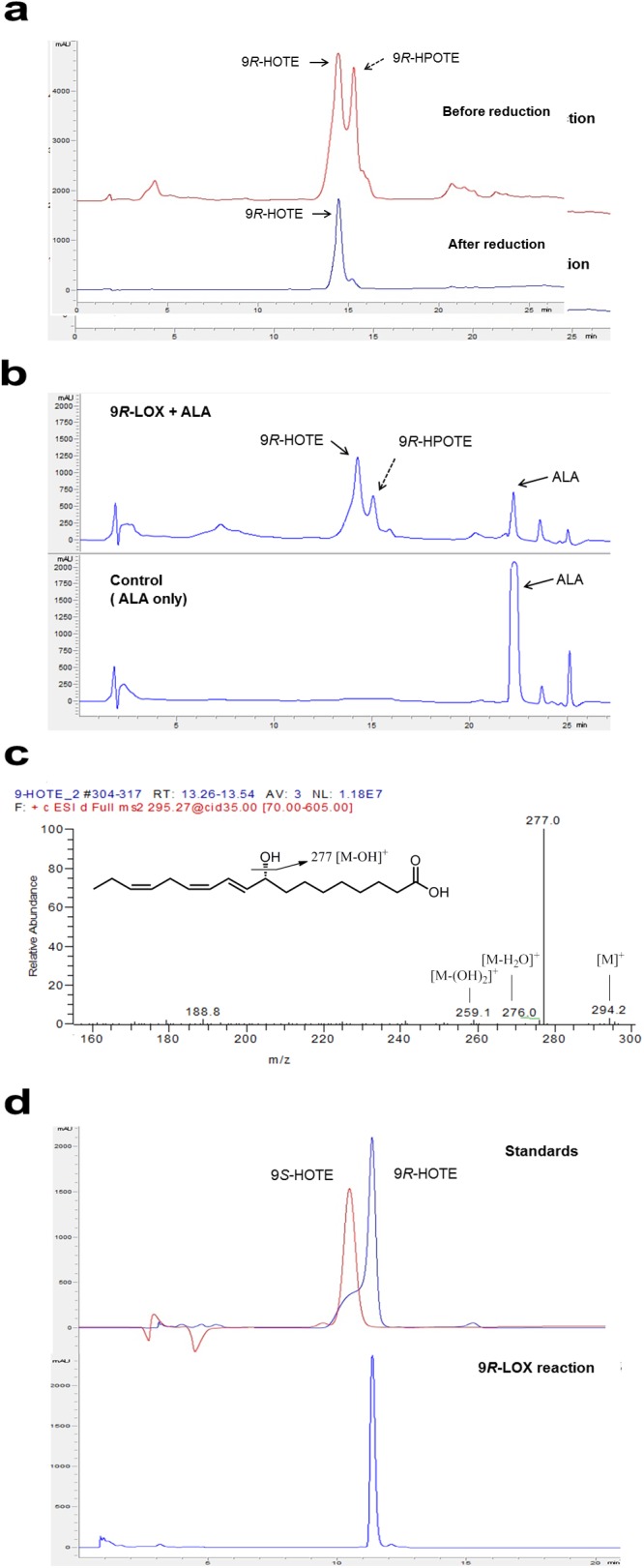
Identification of reaction product of 9*R*-LOX by HPLC and LC-MS/MS. The reaction was performed in 50 mM Tris-HCl buffer (pH 8.5) containing 10 mM ALA, 0.05% Tween 80, and 0.25 U ml^−1^ enzyme at 20°C for 10 min. **a** Reversed-phase HPLC for 9*R*-HPOTE and reduced 9*R*-HOTE formation from ALA. **b** Reversed-phase HPLC for 9*R*-HOTE formation and substrate (ALA) reduction. The control reaction was carried out without the addition of 9*R*-LOX. **c** LC-MS/MS of converted product by 9*R*-LOX. **d** Chiral-phase HPLC for 9*R*-HOTE.

Purified recombinant 9*R*-LOX converted ALA to 9-HPOTE and 9-HOTE, and the formed 9-HPOTE subsequently reduced 9-HOTE by treatment with the reducing agent NaBH_4_, based on reversed-phase HPLC at 234 nm (Fig 2A). After treatment with reducing agent during the same reaction, 9-HOTE (retention time: 13.1 min) and substrate ALA reduction were detected by non-specific measurement at 202 nm (Fig 2B). In the control reaction without the addition of enzyme, no HPFAs or HFAs were formed, indicating that LOX catalyzed the conversion of ALA to 9-HOTE. When the same reaction was carried out with LA, 9-HPODE and 9-HODE were also formed. After treatment with reducing agent, the formed 9-HPODE was reduced to 9-HODE. The retention time of the 9-HODE was 14.3 min, which was different with that of 9-HOTE.

LC-MS/MS analysis was performed to identify the peak obtained from ALA by 9*R*-LOX reaction. The total molecular mass of the product was examined as a peak at *m/z* 294, corresponding to the molecular mass of 9-HOTE in full-scan mode of LC-MS/MS. [19, 22] The peaks at *m*/*z* 277 and *m*/*z* 276 were formed by the loss of OH and H_2_O from the total molecular mass (Fig 2C). Therefore, the peak was identified as 9-HOTE. The product formed with LA was also identified by LC-MS/MS as a total molecular mass peak at *m/z* 295 and *m/z* 171. These peaks were the same as those with 9-HODE from LA and coincide with reported data (S1 Fig) [28]. Since the total mass of 9*S*-HOTE is the same as that of 9*R*-HOTE, stereochemistry of the product was confirmed by HPLC using chiral-phase column chromatography with 9*R*- and 9*S*-HOTE standards. The retention time of the product was the same as that of the 9*R*-HOTE standard (Fig 2D). The 9-HODE product obtained from LA by 9*R*-LOX also showed the same retention time as the 9*R*-HODE standard. Therefore, 9*R*-LOX catalyzed the conversion of 9*R*-HOTE and 9*R*-HODE from ALA and LA, respectively.

### Effects of pH and temperature on the activity of 9*R*-LOX from *Nostoc* sp.

The effect of pH on the activity of 9*R*-LOX from *Nostoc* sp. for the conversion of ALA to 9*R*-HOTE was examined in a pH range from 7.0 to 10.5 (S2A Fig). Maximal activity was observed at pH 8.5. The activity at pH 4.5 and pH 6.5 was approximately 50% of the maximum. The effect of temperature on 9*R*-LOX activity was investigated by varying the temperature from 10 to 45°C at pH 8.5 (S2B Fig). Maximal activity was observed at 15°C. The thermal stability of 9*R*-LOX was examined at temperatures ranging from 15 to 40°C for 3 h (S2C Fig). The activity of 9*R*-LOX was maintained at 100% at temperatures within 15°C. However, above this temperature, the activity decreased with increasing temperature.

### Effects of solvent and detergent on the activity of 9*R*-LOX from *Nostoc* sp.

**Fig 3:**
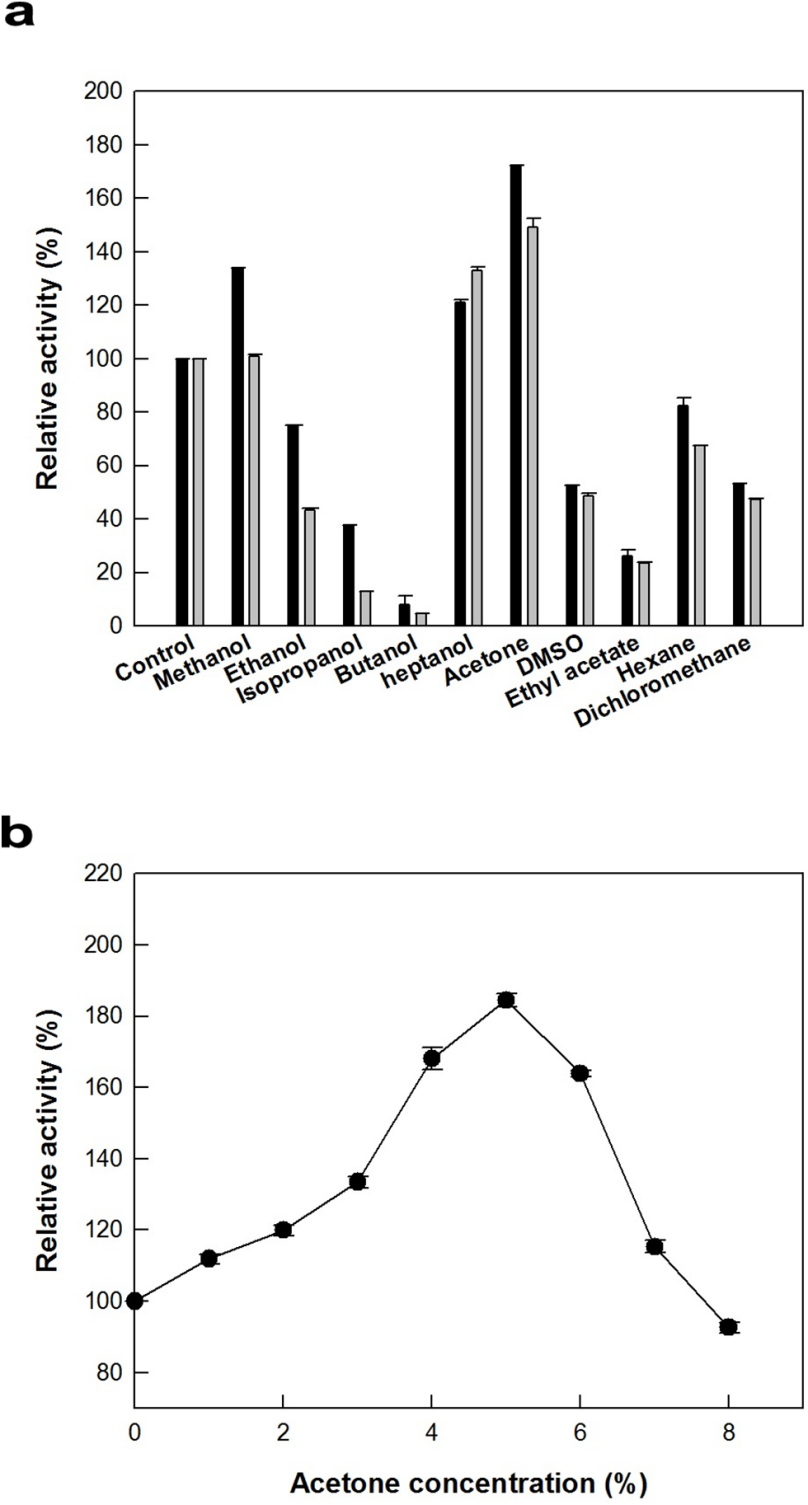
Effect of solvent on the activity of 9*R*-LOX from *Nostoc* sp. The data represent the means of three separate experiments, and error bars represent the standard deviation. **a** Effect of solvent type. The reactions were performed in 50 mM Tris-HCl buffer (pH 8.5) containing 0.25 mM ALA, 0.1 U ml^−1^ enzyme, and 4 (*black bar*) or 6% (*grey bar*) (v/v) of solvent at 15°C for 10 min. **b** Effect of acetone concentration. The reactions were performed in 50 mM Tris-HCl buffer (pH 8.5) containing 0.25 mM ALA and 0.1 U ml^−1^ enzyme by varying the acetone concentration from 1 to 10% (v/v).

**Fig 4:**
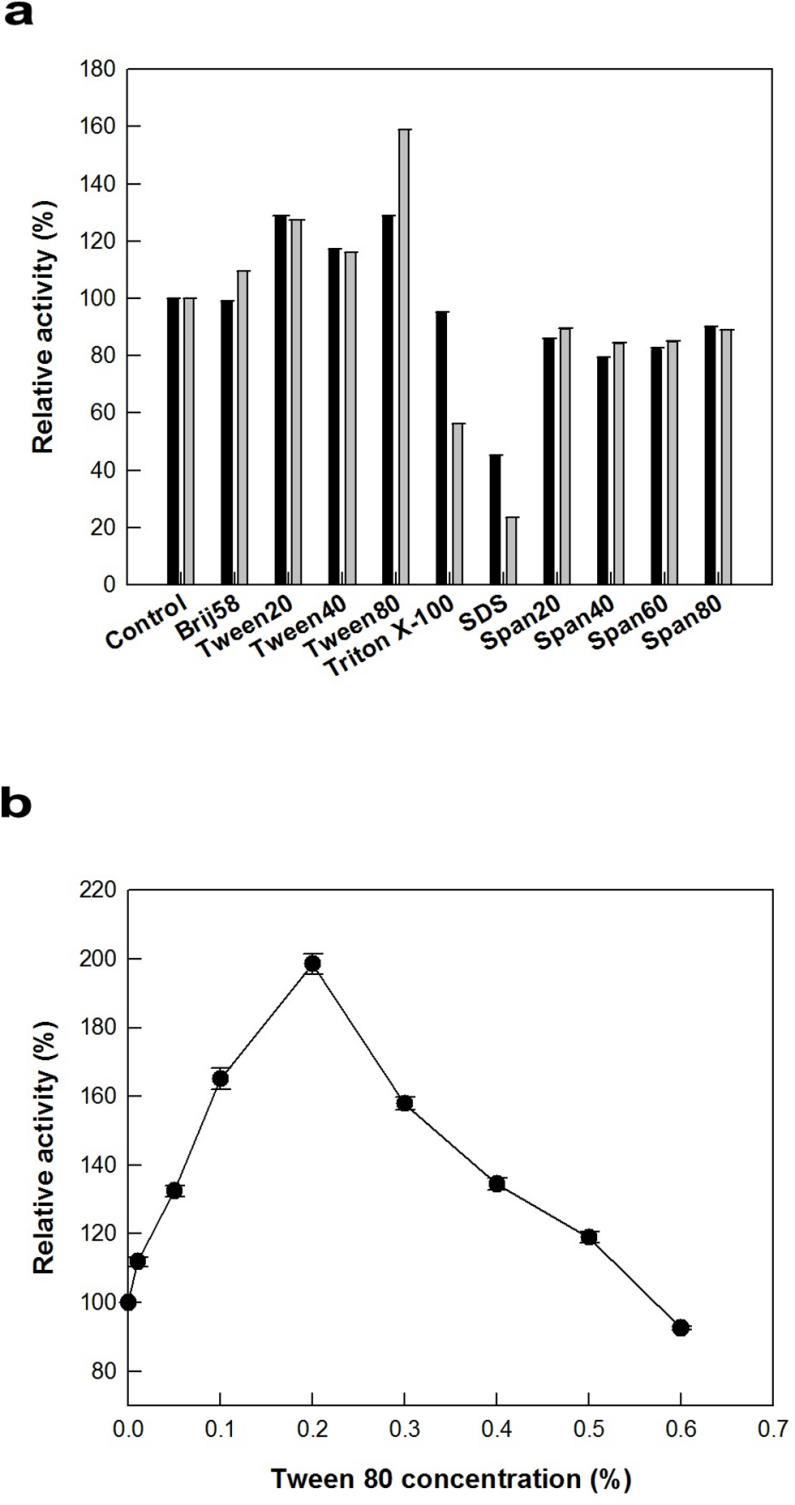
Effect of detergent on the activity of 9*R*-LOX from *Nostoc* sp. The data represent the means of three separate experiments, and error bars represent the standard deviation. **a** Effect of detergent type. The reactions were performed in 50 mM Tris-HCl buffer (pH 8.5) containing 0.25 mM ALA, 0.1 U ml^−1^ enzyme, and 0.02 (*black bar*) or 0.1% (*grey bar*) (w/v) of detergent at 15°C for 10 min. **b** Effect of Tween 80 concentration. The reactions were performed in 50 mM Tris-HCl buffer (pH 8.5) containing 0.25 mM ALA and 0.1 U m^−1^ enzyme by varying the Tween 80 concentration from 0.005 to 1% (w/v) at 15°C for 10 min.

The effect of organic solvent on the activity of 9*R*-LOX from *Nostoc* sp. for 9*R*-HOTE formation was examined at the concentrations of 4% and 6% (v/v). Among the solvents tested, acetone was the most effective solvents for LOX activity (Fig 3A). The effect of acetone concentration on the activity of 9*R*-LOX was evaluated by varying the concentration from 1% to 10% (v/v). The maximum enzyme activity occurred at 5% (v/v) acetone (Fig 3B). The effect of detergent on the activity of 9*R*-LOX for 9*R*-HOTE production was also investigated at concentrations of 0.02% and 0.1% (v/v). Tween 20, Tween 40, and Tween 80 at these concentrations showed 1.2- to 1.6-fold higher activity than that of control (Fig 4A). Tween 80 was the most active detergent for the production of 9*R*-HOTE from ALA. The effect of Tween 80 concentration on the activity of 9*R*-LOX was evaluated by varying the concentration from 0.1% to 0.8% (v/v). The maximum enzyme activity occurred at 0.2% (v/v) Tween 80 (Fig 4B). Solvent and detergent are important sources for the LOX reaction in mixing, dispersing reactant, and providing more chance for the substrate and catalyst to interact. The addition of 5% (v/v) acetone and 0.2% (v/v) Tween 80 was 1.9-fold higher in the conversion activity of 9*R*-LOX than that without the addition of solvent and detergent.

### Substrate specificity of 9*R*-LOX from *Nostoc* sp.

LOX activity was optimal under the conditions of 5% (v/v) acetone and 0.2% (w/v) Tween 80 at pH 8.5 and 15°C for 5 min. Under these conditions, the specific activity and Michaelis−Menten constants (*K*_m_), turnover numbers (*k*_cat_), and catalytic efficiencies (*k*_cat_/*K*_m_) of *Nostoc* sp. 9*R*-LOX were investigated using PUFAs, including LA, ALA, and GLA (Table 1 and S3 Fig). The specific activity of the enzyme followed the order LA > ALA > GLA with 18.4, 6.3, and 5.4 U mg^−1^, respectively. The specific activity of *Nostoc* sp. 9*R*-LOX for ALA was lower than that for LA. The catalytic efficiencies of *Nostoc* sp. 9*R*-LOX for LA, ALA and GLA were 507, 137, and 109 mM^−1^ min^−1^, respectively, and their order was the same as that observed for the specific activity.

**Table 1:** Specific activity and kinetic parameters of recombinant *Nostoc* sp. 9*R*-LOX for PUFAs.

Substrate	Product	Specific activity (U mg^−1^)	*K*_m_ (μM)	*k*_cat_ (1/min)	*k*_cat_/*K*_m_ (mM^−1^ min^−1^)
Linoleic acid	9*R*-HODE	18.4 ± 1.1	404 ± 30	307 ± 8	760 ± 8
α-Linolenic acid	9*R*-HOTE (9*Z*,12*E*,15*Z*)	6.3 ± 0.4	54 ± 1.3	7.8 ± 0.2	144 ± 3
γ-Linolenic acid	9*R*-HOTE (6*Z*,10*E*,12*Z*)	5.4 ± 0.6	54 ± 0.8	5.8 ± 0.4	109 ± 7

HODE: hydroxyoctadecadienoic acid, HOTE: hydroxyoctadecatrienoic acid

### Optimization of reaction conditions for the increased production of 9*R*-HOTE from ALA by 9*R*-LOX from *Nostoc* sp.

**Fig 5:**
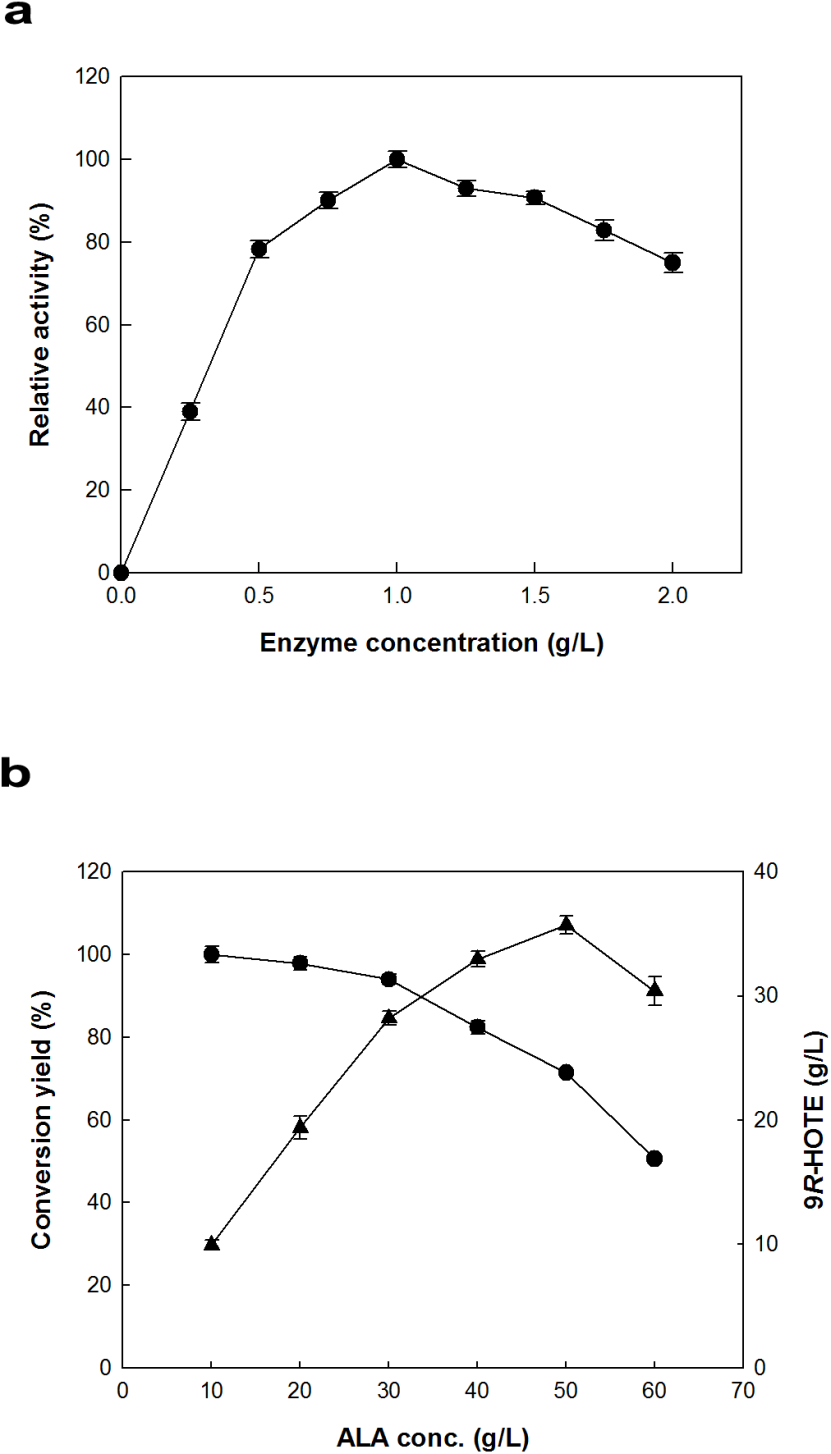
Effects of the concentrations of enzyme and substrate on the production of 9*R*-HOTE from ALA by 9*R*-LOX. The data represent the means of three separate experiments, and error bars represent the standard deviation. **a** Effect of enzyme concentration. The reactions were performed in 50 mM Tris-HCl buffer (pH 8.5) containing 10 g l^−1^ ALA, 5% (v/v) acetone, and 0.2% (w/v) Tween 80 at 15°C for 20 min by varying the enzyme concentration from 0.05 to 2 g l^−1^. **b** Effect of ALA concentrations. The reactions were performed in 50 mM Tris-HCl buffer (pH 8.5) containing 0.5 g l^−1^ enzyme, 5% (v/v) acetone, and 0.2% (w/v) Tween 80 at 15°C for 20 min by varying the ALA concentrations from 10 to 60 g l^−1^.

To determine the maximal production of 9*R*-HOTE from ALA using *Nostoc* sp. 9*R*-LOX, the enzyme concentration was investigated by varying the enzyme concentration from 0.25 to 2 g/L in the presence of 5% (v/v) acetone and 0.2% (w/v) Tween 80 at pH 8.5 and 15°C. The maximum production of 9*R*-HOTE was observed at 1.0 g/L enzyme (Fig 5A). The concentration of ALA for the maximum production of 9*R*-HOTE was also investigated by varying the concentration of ALA as a substrate from 10 to 60 g/L using 1 g/L enzyme in the presence of 5% (v/v) acetone and 0.2% (w/v) Tween 80 at pH 8.5 and 15°C (Fig 5B). At concentrations below 50 g/L ALA, 9*R*-HOTE production was increased with increasing ALA concentration. However, the conversion yield decreased as the concentration of substrate increased. Therefore, a substrate concentration of 40 g/L ALA for 9*R*-HOTE production was selected as a suitable concentration for improving the performance in terms of both conversion yield and product concentration.

### Preparation of PO hydrolyzate by CRL and its fatty acid composition

**Fig 6:**
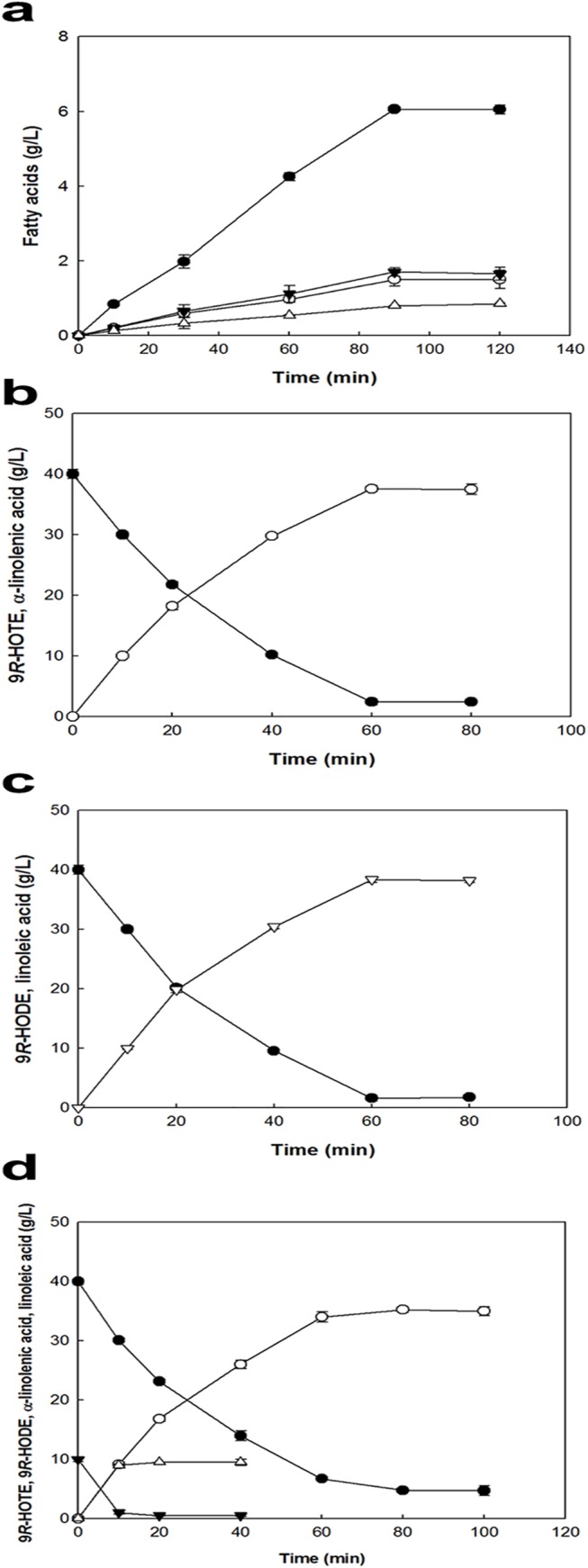
Time-course reactions for the production of fatty acids by CRL, 9*R*-HOTE and 9*R*-HODE by recombinant 9*R*-LOX. The data represent the means of three separate experiments, and error bars represent the standard deviation. **a** Production of fatty acids from PO by CRL. Fatty acids were α-linoleic acid (*filled circle*), linoleic acid (*empty circle*), oleic acid (*filled inverse triangle*), and other fatty acids (*empty triangle*). The reactions were performed in 50 mM Tris-HCl buffer (pH 7.5) containing 1 g l^−1^ CRL, 10 g l^−1^ PO, and 0.02% (w/v) Tween 80 at 37°C with agitation at 200 rpm for 2 h. **b** Production of 9*R*-HOTE (*empty circle*) and 9*R*-HODE (*empty triangle*) from ALA (*filled circle*) and LA (*filled inverse triangle*) in PO hydrolyzate. The reactions were performed in a 500 mL baffled flask in 80 mL of 50 mM Tris-HCl buffer (pH 8.5) consisting of PO hydrolyzate, which contained 40 g l^−1^ ALA, 10 g l^−1^ LA, 15.3 g l^−1^ of other fatty acids, 5% (v/v) acetone, 0.2% (v/v) Tween 80, and 1 g l^−1^ purified 9*R*-LOX at 15°C for 100 min with agitation at 250 rpm. **c** Production of 9*R*-HOTE (*empty circle*) from reagent grade ALA (*filled circle*) by 9*R*-LOX. The reactions were performed in 50 mM Tris-HCl buffer (pH 8.5) containing 40 g/L reagent-grade ALA, 1 g l^−1^ 9*R*-LOX, 5% (v/v) acetone, and 0.2% (w/v) Tween 80 at 15°C for 80 min. **d** Production of 9*R*-HODE (*empty inverse triangle*) from reagent-grade LA (*filled circle*) by 9*R*-LOX. The reactions were performed in the same manner as those of 9*R*-HOTE production, except for the substrate LA.

Natural PO was hydrolyzed by CRL because *Nostoc* sp. 9*R*-LOX cannot direct the hydroxylation of PO. ALA from PO was prepared by the hydrolysis of PO using CRL, because CRL exhibited the highest activity for releasing free ALA from PO among the lipases tested (S4 Fig). The hydrolytic reaction for the oil was performed with 10 g/L PO and 1 g/L CRL at pH 7.5, 35°C, and 250 rpm for 120 min. At 90 min, PO hydrolyzate contained 6 g/L ALA, 1.5 g/L LA, 1.7 g/L oleic acid (OA), and 0.8 g/L saturated fatty acids (Fig 6A). After 90 min, the contents of these fatty acids decreased with increasing reaction time due to reverse reaction. Therefore, the reaction was terminated at 90 min, and the resulting composition is presented in S1 Table. The obtained PO hydrolyzate contained 60% ALA and 15% LA, and its composition was similar to that obtained the previously published data [15]. Therefore, a total of 75% substrates (ALA and LA) in PO hydrolyzate can be utilized for 9*R*-HOTE and 9*R*-HODE production using 9*R*-LOX.

### Time-course reactions for the production of 9*R*-HOTE and 9*R*-HODE from ALA and LA by 9*R*-LOX from *Nostoc* sp.

The optimal conditions for 9*R*-HOTE conversion of the recombinant purified 9*R*-LOX from *Nostoc* sp. were pH 8.5, 15°C, 1 g/L enzyme, 40 g/L reagent ALA, 5% (v/v) acetone, and 0.02% (w/v) Tween 80 with agitation speed of 250 rpm and oxygen at 20 ml of working volume in a 250 ml-baffled flask. Under the optimal conditions, the production of 9*R*-HOTE and 9*R*-HODE from PO hydrolyzate containing ALA and LA were performed using 9*R*-LOX. The enzyme produced 36.5 g/L of 9*R*-HOTE and 9.5 g/L of 9*R*-HODE from 40 g/L of ALA and 10 g/L of LA in PO hydrolyzate for 100 min. 9*R*-HOTE production from ALA in PO hydrolyzate was increased to 34 g/L at 60 min and plateaued at 35 g/L at 80 min. The conversion yield and volumetric productivity for 9*R*-HOTE from ALA in PO hydrolyzate were 85% and 34 g/L/h, respectively. 9*R*-HODE production from LA in PO hydrolyzate was more rapid than that from ALA and plateaued at 20 min with a conversion yield of 95% (Fig 6B). 9*R*-LOX produced 37.6 g/L 9*R*-HOTE from 40 g/L of reagent-grade ALA at 60 min, with a conversion yield of 94%, a molar yield of 89%, and a volumetric productivity of 37.6 g/L/h (Fig 6C). Since the 9*R*-LOX activity for LA was highest, the enzymatic conversion of 9*R*-HODE from reagent grade LA was also performed under the same conditions. 9*R*-LOX produced 38.4 g/L 9*R*-HODE from 40 g/L LA at 60 min, with a conversion yield of 96%, a molar yield of 91%, and a volumetric productivity of 38.4 g/L/h (Fig 6D). These results are the first data for showing the enzymatic production of 9*R*-HOTE and 9*R*-HODE from not only reagent grade ALA and LA but also ALA and LA in PO hydrolyzate using LOX (Table 2).

**Table 2:** Production of HPFA and HFA from PUFA and oil hydrolyzate by LOX.

Source of LOX	Biocatalyst	Substrate (g/L)	Product (g/L)	Conversion yield (%)	Productivity (g/L/h)	References
potato	immobilized	LA (0.12)	9-HODE (0.077)	64.2	NA	[39]
potato	crude	TLH (1.5)	9*S*-HPODE (1.24)	82.7	NA	[40]
soybean	two-phase	LA (20)	13-HPODE* (12.04)	60.2	NA	[33]
soybean	crude	LA (40)	13*S*-HPODE (36)	90	9	[34]
soybean	crude	TLH (6**)	13*S*-HPODE (5.7)	96.1	NA	[35]
soybean	crude	FOH (78.5***)	13*S*-HPOTE**** (62.8)	80	31.4	[36]
soybean	partial purified	LA (28)	13-HPODE (22.4)	80	22.4	[37]
soybean	partial purified	LA (28)	13*S*-HPODE (15.1)	54	NA	[38]
*G*. *graminis tritici* LOX in *T*. *reesei*	secreted crude	LA (300)	13*S*-HPODE* (120)	40	3.6	[42]
*B thaliandensis* LOX in *E*. *coli*	Recombinant purified enzyme	LA (20)	13S-HODE (20.8)	98.5	10.4	[31]
*P*. *aeruginose* LOX in *T*. *reesei*	secreted crude	LA (10)	9*S*- and 13*S*-HPODE (7.5)	75	0.16	[41]
*Nostoc* sp. LOX in *E*. *coli*	Recombinant whole cell	LA (15)	9*R*-HODE (14.3)	95	14.3	[23]
*Nostoc* sp. LOX in *E*. *coli*	Recombinant purified enzyme	LA(40)	9*R*-HODE(38.4)	96	38.4	This study
		ALA(40)	9*R*-HOTE(37.6)	94	37.6	This study
		POH (40***)	9*R*-HOTE (35.2)	85	34	This study

NA: Not available, TLH: Trilinolein hydrozate, FOH: Flax seed oil hydrozate, POH: Perilla seed oil hydrozate.

* *E*,*Z*-form only

** LA only

*** ALA only

**** *E*,*Z*,*Z*-form only.

## Discussion

The characterization and application of bacterial LOX emerged only last decades. Recent investigations revealed that bacterial LOX possessed different properties including unusual stereo-selectivity as well as substrate specificity and molecular size, comparing with plant and animal LOXs. Dimeric form comprising N-terminal domain and the catalytic β-barrel domain is common in LOX but the molecular masses of the reported bacterial LOXs were differently ranged as a mainly 44−65 kDa [6, 26], comparing with a 70–80 kDa in fungi [7], 94−104 kDa dimer in plants, and 75−80 kDa monomer in animals [3, 29]. *Nostoc* sp. recombinant 9*R*-LOX also showed short N-terminal domain and catalytic domain with 52 kDa of molecular mass in denatured form, however, native form of associated subunit is firstly identified as a trimeric LOX in this study (Fig 1).

The substrate specificity of bacterial LOXs toward omega-3 PUFAs including ALA based on kinetic parameters is also noteworthy properties. The kinetic parameters of bacterial LOXs can be determined by not only an O_2_ probe method but also a spectrophotometry method. However, it is difficult to measure the activities at the high concentrations of substrate and enzyme using the spectrophotometry method. Moreover, the spectrophotometry method is less accurate. Therefore, only O_2_ probe method was used in the present study for the determination of the kinetic parameters of LOX. The kinetic parameters of the known microbial LOX enzymes for ALA have been reported only in LOXs from *Acaryochloris marina*, *Burkholderia thailandensis*, and *Fusarium oxysporum*, which produce 12*R*-HOTE and 13*S*-HOTE from ALA [7, 30, 31]. The *k*_cat_/*K*_m_ of LOXs from *A*. *marina*, *B*. *thailandensis*, and *F*. *oxysporum* for ALA were 0.41, 0.042, and 0.032 mM/min, respectively. Catalytic efficiency of *Nostoc* 9*R*-LOX for ALA was 205 mM/min and, therefore, was highest among the known microbial LOX owing to its high substrate affinity for PUFA (Table 1). Therefore, recombinant *Nostoc* 9*R*-LOX was successfully applied for regio- and stereo-specific conversion of 9*R*-HOTE from ALA in this study (Fig 2). 9*R*-ConFig.uration of hydroxyl fatty acid was rarely observed in a few organisms, including cyanobacteria [32] and hydra [22], comparing with *S*-configuration of 9-hydroxy fatty acids derived from other LOXs. 9-HOTE from ALA is known as a signaling molecule [20]. Moreover, ALA is further converted eicosapentanoic acid (20:5, *n*-3) and docosahexanoic acid (22:6, *n*-3), the important precursors of human signaling cascades [2]. However, its practical application of omega-3 PUFAs for signaling or renewable purpose were limited owing to the lack of high and specific LOXs toward omega-3 PUFA as well as appropriate optimization including supplementation of oxygen, solvent, detergent, enzyme, and substrates.

The production of HPFAs and HFAs is summarized in Table 2. Soybean LOX (SLOX) was mainly used for the enzymatic production of 13*S*-HPODE from LA by the crude extract or the partially purified enzyme conversion, immobilization, biphasic reaction [33–38]. Crude extract of SLOX produced 5–62 g/L with 54–96% conversion yield of 13-HPODE from LA. Alternatively, potato LOX has also been used for production of 9*S*-HPODE or 9*S*-HODE from LA using crude extract or by immobilization [39, 40]. A few reports showed HPODE production from vegetable oil. Trilinolein hydrolyzate (TLH) containing LA and flax seed oil hydrolyzate (FOH) containing ALA were used for the production of 13*S*-HPODE and 13*S*-HPOTE, respectively. SLOX produced 5.7 g/L 13*S*-HPODE from 6 g/L LA in TLH with a conversion yield of 96% [35]. SLOX produced the 10*E*,12*Z*,15*Z*-form of 13*S*-HPOTE from 78.5 g/L ALA in FOH with a conversion yield of 62.8% and a productivity of 31.4 g/L/h [36]. Fungal LOX such as *Gaeumannomyces graminis tritici* LOX secreted in *Trichoderma reesei* showed a conversion yield of 40% and a productivity of 3.6 g/L^/^h for 10*E*,12*Z*-13*S*-HPOTE production [41]. A LOX from *Pseudomonas aeruginosa* that was cloned and secreted in *Trichoderma reesei* showed mixed production of 9*S*- and 13*S*-HPODE from LA with a low yield [42]. Recently, recombinant *Burkholderia thailandensis* LOX showed 10.4 g/L/h of productivity with a molar conversion yield of 98.5% [29].

In the present study, recombinant purified 9*R*-LOX from *Nostoc* sp. produced 9*R*-HOTE with a higher conversion yield and productivity from ALA in PO hydrolyzate as well as reagent-grade ALA than those of other LOXs for PUFAs (Table 2, Fig 6B and 6C). 9*R*-LOX also converted 9*R*-HODE from reagent grade LA with the highest yield and productivity ever reported (Table 2 and Fig 6D). As shown in Table 1, the catalytic efficiency of 9*R*-LOX for LA was 5.3-fold higher than that for ALA, indicating that LA is a better substrate than ALA. If LA-rich vegetable oil is used, the production of HFA by 9*R*-LOX from *Nostoc* sp. will increase. The typical LA-rich vegetable oil is safflower oil, which is consisted of 80% LA, 14% oleic acid, and 5% palmitic acid. The present study is focused on the stereo-selective enzymatic production of 9*R*-HOTE. However, safflower oil was not used for 9*R*-HOTE because it contained little ALA. Thus, the LA-rich vegetable oil was not applied to the production of HFA by 9*R*-LOX from *Nostoc* sp. Existing sources and methodology for the production of 9-hydroxy fatty acid relied on LOX from only plant source such as potato. However, plant source LOX cannot be applied to 9-HODE production because of its low yield. Therefore, 9*R*-LOX is an alternative and efficient biocatalyst for 9*R*-HOTE and 9*R*-HODE production from reagent grade ALA and LA as well as LA- and ALA-rich vegetable oil.

HFAs originating from the LOX reaction exhibited a variety of bioactivities including antifungal and immune modulation activity [19, 43]. Thus, the production and application of HFAs from ALA using LOX is interesting for further understanding of signaling cascades. Vegetable oils are widely utilized in the human diet and in many other industrial applications [44]. Furthermore, a large volume of surplus vegetable oil is currently an important issue [45]. The PUFA component in vegetable oils provides an opportunity to manufacture value-added products such as HFAs, green notes, and lactones. ALA and LA are one of the major components in vegetable oils including PO, flax seed oil, and chia oil, which contain over 60% ALA. However, except for LA, the current LOXs are limited in their practical utilization of *n*-3 or *n*-6 PUFAs. Therefore, our study offers a new opportunity for the biotechnological production of value added product from vegetable oil using recombinant LOX, by the efficient utilization of PUFAs in vegetable oil.

## Conclusion

In conclusion, the production of 9*R*-HOTE from ALA in PO was demonstrated through enzymatic hydrolysis and oxygenation processes. CRL-treated PO hydrolyzate containing ALA and LA was converted into 9*R*-HOTE and 9*R*-HODE by purified recombinant 9*R*-LOX. To the best of our knowledge, our enzymatic conversion exhibits the highest productivity and yield of 9*R*-HOTE and 9*R*-HODE from ALA and LA in natural oil by recombinant LOX, respectively, reported up to date. Thus, the enzymatic production of 9*R*-HOTE from PUFA of vegetable oil may be useful for efficient oxyfunctionalization and value-added product biotransformation.

## Supporting Information

S1 Fig LC-MS/MS analysis of 9*R*-HODE from LA by 9*R*-LOX. The reaction was performed in 50 mM Tris-HCl buffer (pH 8.5), 10 mM LA, and 0.25 U ml^−1^ enzyme at 25°C for 5 min.
(TIF)

S2 Fig Effects of pH, temperature, and stability on the activity of 9*R*-LOX from *Nostoc* sp. The data represent the means of three separate experiments, and error bars represent the standard deviation. **a** Effect of pH. The reactions were performed by varying the pH from 7.0 to 10.0 using 50 mM Tris-HCl buffer (pH 7.0–9.0) (*filled circle*) and 50 mM sodium borate buffer (pH 9.0–10.0) (*empty circle*) containing 0.25 mM ALA and 0.05 U/mL enzyme at a constant temperature of 25°C for 5 min. **b** Effect of temperature. The reactions were performed by varying the temperature from 10 to 50°C in 50 mM Tris-HCl buffer (pH 8.5) containing 0.25 mM ALA and 0.05 U ml^−1^ enzyme for 5 min. **c** The effect of temperature on 9*R*-LOX stability. Experiments were carried out after incubation at temperatures ranging from 4 to 45°C for 4 h. Samples were withdrawn at time intervals and then assayed in 50 mM Tris-HCl buffer (pH 8.5) containing 0.25 mM ALA and 0.05 U ml^−1^ enzyme at 15°C for 5 min.
(TIF)

S3 Fig Hanes-Woolf plot (S vs S/V) from the Michaelis-Menten equation of 9*R*-LOX from *Nostoc* sp. for each unsaturated fatty acid substrate. **a** Reacted with LA. **b** Reacted with ALA. **c** Reacted with GLA.
(TIF)

S4 Fig Activities of lipases for releasing ALA from PO. The reactions were performed in 50 mM Tris-HCl buffer (pH 7.5), 1 U ml^−1^ lipase, and 0.1 g^−1^ L^−1^ of PO at 30°C for 30 min. Recombinant AOL, CRL, TLL, RML, PCL, and PFL were used for the hydrolysis of PO.
(TIF)

S1 Table Composition of fatty acids for PO hydrolyzate. (DOCX)

## References

[1] Ivanov I, Heydeck D, Hofheinz K, Roffeis J, O’donnell VB, et al Molecular enzymology of lipoxygenases. Arch Biochem Biophys. 2010;503(2): 161–174. 10.1016/j.abb.2010.08.016 20801095

[2] Joo YC, Oh DK. Lipoxygenases: Potential starting biocatalysts for the synthesis of signaling compounds. Biotechnol Adv. 2012;30(6): 1524–1532. 10.1016/j.biotechadv.2012.04.004 22537875

[3] Brash AR. Lipoxygenases: Occurrence, functions, catalysis, and acquisition of substrate. J Biol Chem. 1999;274(34): 23679–23682. 10446122 10.1074/jbc.274.34.23679

[4] Samuelsson B, Dahlen S, Lindgren J, Rouzer C, Serhan C. Leukotrienes and lipoxins: structures, biosynthesis, and biological effects. Science. 1987;237(4819): 1171–1176. 2820055 10.1126/science.2820055

[5] Su C, Oliw EH. Manganese lipoxygenase: purification and characterization. J Biol Chem. 1998;273(21): 13072–13079. 9582345 10.1074/jbc.273.21.13072

[6] Zheng Y, Boeglin WE, Schneider C, Brash AR. A 49-kDa mini-lipoxygenase from *Anabaena* sp. PCC 7120 retains catalytically complete functionality. J Biol Chem. 2008;283(8): 5138–5147. 18070874 10.1074/jbc.M705780200

[7] Brodhun F, Cristobal-Sarramian A, Zabel S, Newie J, Hamberg M, et al An Iron 13*S*-Lipoxygenase with an α-Linolenic Acid Specific Hydroperoxidase Activity from *Fusarium oxysporum* . PLoS ONE. 2013;8(5): e64919 10.1371/journal.pone.0064919 23741422 PMC3669278

[8] Noordermeer MA, van der Goot W, van Kooij AJ, Veldsink JW, Veldink GA, et al Development of a biocatalytic process for the production of C6-aldehydes from vegetable oils by soybean lipoxygenase and recombinant hydroperoxide lyase. J Agric Food Chem. 2002;50(15): 4270–4274. 12105957 10.1021/jf0202685

[9] Wang X, Lu F, Zhang C, Lu Y, Bie X, et al Effects of recombinated *Anabaena* sp. lipoxygenase on the protein component and dough property of wheat flour. J Agric Food Chem. 2014. 10.1021/jf503238h 25247399

[10] Zhang C, Tao T, Ying Q, Zhang D, Lu F, et al Extracellular production of lipoxygenase from *Anabaena* sp. PCC 7120 in *Bacillus subtilis* and its effect on wheat protein. Appl Microbiol Biotechnol. 2012;94(4): 949–958. 10.1007/s00253-012-3895-5 22290650

[11] Marques G, Molina S, Babot ED, Lund H, Río JCd, et al Exploring the potential of fungal manganese-containing lipoxygenase for pitch control and pulp delignification. Bioresour Technol. 2011;102(2): 1338–1343. 10.1016/j.biortech.2010.08.112 20864336

[12] Kim KR, Oh DK. Production of hydroxy fatty acids by microbial fatty acid-hydroxylation enzymes. Biotechnol Adv. 2013;31(8): 1473–1485. 10.1016/j.biotechadv.2013.07.004 23860413

[13] Hou CT. Biotransformation of unsaturated fatty acids to industrial products. Adv Appl Microbiol. 47: Academic Press; 2000 pp.201–220. 12876798 10.1016/s0065-2164(00)47005-x

[14] Jo YS, An JU, Oh DK. γ-Dodecelactone production from safflower oil via 10-hydroxy-12(*Z*)-octadecenoic acid intermediate by whole cells of *Candida boidinii* and *Stenotrophomonas nitritireducens* . J Agric Food Chem. 2014;62(28): 6736–6745. 10.1021/jf501081z 24967938

[15] Ciftci ON, Przybylski R, Rudzińska M. Lipid components of flax, perilla, and chia seeds. Eur J Lipid Sci Technol. 2012;114(7): 794–800.

[16] Shin HS, Kim SW. Lipid composition of perilla seed. J Am Oil Chem Soc. 1994;71(6): 619–622.

[17] Okuno M, Kajiwara K, Imai S, Kobayashi T, Honma N, et al Perilla oil prevents the excessive growth of visceral adipose tissue in rats by down-regulating adipocyte differentiation. J Nutr. 1997;127(9): 1752–1757. 9278555 10.1093/jn/127.9.1752

[18] Shimura M, Mase S, Iwata M, Suzuki A, Watanabe T, et al Anti-conidial germination factors Induced in the presence of probenazole in infected host leaves. III. Structural elucidation of substances A and C. Agric Biol Chem. 1983;47(9): 1983–1989.

[19] Dong M, Oda Y, Hirota M. (10*E*,12*Z*,15*Z*)-9-Hydroxy-10,12,15-octadecatrienoic acid methyl ester as an anti-inflammatory compound from *Ehretia dicksonii* . Biosci Biotechnol Biochem. 2000;64(4): 882–886. 10830513 10.1271/bbb.64.882

[20] Obinata H, Hattori T, Nakane S, Tatei K, Izumi T. Identification of 9-hydroxyoctadecadienoic acid and other oxidized free fatty acids as ligands of the G protein-coupled receptor G2A. J Biol Chem. 2005;280(49): 40676–40683. 16236715 10.1074/jbc.M507787200

[21] Akakabe Y, Matsui K, Kajiwara T. Enantioselective formation of (*R*)-9-HPODE and (*R*)-9-HPOTrE in marine green alga *Ulva conglobata* . Bioorg Med Chem. 2002;10(10): 3171–3173. 12150862 10.1016/s0968-0896(02)00212-2

[22] Marzo V, Vardaro RR, Petrocellis L, Cimino G. ω10-Lipoxygenase products of α-linolenic acid are esterified to phospholipids in *Hydra vulgaris* . Experientia. 1996;52(2): 120–126.

[23] Kim KR, Seo MH, Park JB, Oh DK. Stereospecific production of 9*R*-hydroxy-10*E*,12*Z*-octadecadienoic acid from linoleic acid by recombinant *Escherichia coli* cells expressing 9*R*-lipoxygenase from *Nostoc* sp. SAG 25.82. J Mol Catal B: Enzym. 2014;104(0): 56–63.

[24] Knapp MJ, Klinman JP. Kinetic studies of oxygen reactivity in soybean lipoxygenase-1. Biochem. 2003;42(39): 11466–11475. 14516198 10.1021/bi0300884

[25] Axelrod B, Cheesbrough TM, Laakso S, John ML. Lipoxygenase from soybeans. Methods Enzymol. Volume 71: Academic Press; 1981 pp.441–451.

[26] KÜHn H, Wiesner R, Alder L, Schewe T. Occurrence of free and esterified lipoxygenase products in leaves of *Glechoma hederacea* L. and other Labiatae. Eur J Biochem. 1989;186(1–2): 155–162. 2598926 10.1111/j.1432-1033.1989.tb15190.x

[27] O'Fallon JV, Busboom JR, Nelson ML, Gaskins CT. A direct method for fatty acid methyl ester synthesis: Application to wet meat tissues, oils, and feedstuffs. J Anim Sci. 2007;85(6): 1511–1521. 17296772 10.2527/jas.2006-491

[28] Berry H, Débat H, Garde VL. Oxygen concentration determines regiospecificity in Soybean lipoxygenase-1 reaction via a branched kinetic scheme. J Biol Chem. 1998;273(5): 2769–2776. 9446584 10.1074/jbc.273.5.2769

[29] Hansen J, Garreta A, Benincasa M, Fusté MC, Busquets M, et al Bacterial lipoxygenases, a new subfamily of enzymes? A phylogenetic approach. Appl Microbiol Biotechnol. 2013;97(11): 4737–4747. 10.1007/s00253-013-4887-9 23624657

[30] Gao B, Boeglin WE, Brash AR. Omega-3 fatty acids are oxygenated at the n-7 carbon by the lipoxygenase domain of a fusion protein in the cyanobacterium *Acaryochloris marina* . Biochim Biophys Acta. 2010;1801(1): 58–63. 10.1016/j.bbalip.2009.09.004 19786119 PMC2787963

[31] An JU, Kim BJ, Hong SH, Oh DK. Characterization of an omega-6 linoleate lipoxygenase from *Burkholderia thailandensis* and its application in the production of 13-hydroxyoctadecadienoic acid. Appl Microbiol Biotechnol. 2015: 1–11. 10.1007/s00253-014-6353-8 25586578

[32] Murakami N, Shirahashi H, Nagatsu A, Sakakibara J. Two unsaturated 9*R*-hydroxy fatty acids from the cyanobacterium *Anabaena flos-aquae f*. *flos-aquae* . Lipids. 1992;27(10): 776–778.

[33] Drouet P, Thomas D, Legoy MD. Production of 13(*S*)-hydroperoxy-9(*Z*),11(*E*)-octadecadienoic acid using soybean lipoxygenase 1 in a biphasic octane-water system. Tetrahedron Lett. 1994;35(23): 3923–3926.

[34] Elshof MBW, Veldink GA, Vliegenthart JFG. Biocatalytic hydroxylation of linoleic acid in a double-fed batch system with lipoxygenase and cysteine. Lipid / Fett. 1998;100(6): 246–251.

[35] Gargouri M, Legoy MD. Bienzymatic reaction for hydroperoxide production in a multiphasic system. Enzyme Microb Technol. 1997;21(2): 79–84.

[36] Fauconnier ML, Marlier M. An efficient procedure for the production of fatty acid hydroperoxides from hydrolyzed flax seed oil and soybean lipoxygenase. Biotechnol Tech. 1996;10(11): 839–844.

[37] Iacazio G, Langrand G, Baratti J, Buono G, Triantaphylides C. Preparative, enzymic synthesis of linoleic acid (13*S*)-hydroperoxide using soybean lipoxygenase-1. J Org Chem. 1990;55(5): 1690–1691.

[38] Martini D, Iacazio G, Ferrand D, Buono G, Triantaphylides C. Optimization of large scale preparation of 13-(*S*)-hydroperoxy-9*Z*,11*E*-octadecadienoic acid using Soybean lipoxygenase. Application to the chemoenzymatic synthesis of (+)-coriolic acid. Biocatalysis. 1994;11(1): 47–63.

[39] del Carmen Pinto M, Gata JL, Macías P. Immobilization of Potato tuber lipoxygenase on oxirane acrylic beads. Biotechnol Prog. 1997;13(4): 394–398. 9265777 10.1021/bp9700465

[40] Gargouri M, Dominique Legoy M. A two-enzyme system for the transformation of unsaturated oils to 9(*S*)-hydroperoxy fatty acids. Biotechnol Lett. 2002;24(11): 915–918.

[41] Villaverde JJ, van der Vlist V, Santos SAO, Haarmann T, Langfelder K, et al Hydroperoxide production from linoleic acid by heterologous *Gaeumannomyces graminis tritici* lipoxygenase: Optimization and scale-up. Chem Eng J. 2013;217(0): 82–90.

[42] Villaverde JJ, Santos SAO, Haarmann T, Neto CP, Simões MMQ, et al Cloned *Pseudomonas aeruginosa* lipoxygenase as efficient approach for the clean conversion of linoleic acid into valuable hydroperoxides. Chem Eng J. 2013;231(0): 519–525.

[43] Namai T, Kato T, Yamaguchi Y, Hirukawa T. Anti-rice Blast Activity and Resistance Induction of C-18 Oxygenated Fatty Acids. Biosci Biotechnol Biochem. 1993;57(4): 611–613. 10.1271/bbb.57.283 27314783

[44] Biermann U, Bornscheuer U, Meier MAR, Metzger JO, Schäfer HJ. Oils and fats as renewable raw materials in chemistry. Angew Chem Int Ed. 2011;50(17): 3854–3871. 10.1002/anie.201002767 21472903

[45] Hou CT. Biotechnology for fats and oils: new oxygenated fatty acids. New Biotechnol. 2009;26(1–2): 2–10. 10.1016/j.nbt.2009.05.001 19447212

