# Supplementary figures and images for: Selective Production of 9R-Hydroxy-10E,12Z,15Z-Octadecatrienoic Acid from α-Linolenic Acid in Perilla Seed Oil Hydrolyzate by a Lipoxygenase from Nostoc Sp. SAG 25.82
Source: PLoS One. 2015 Sep 17;10(9):e0137785. doi: 10.1371/journal.pone.0137785 (PMC4574779; doi:10.1371/journal.pone.0137785)

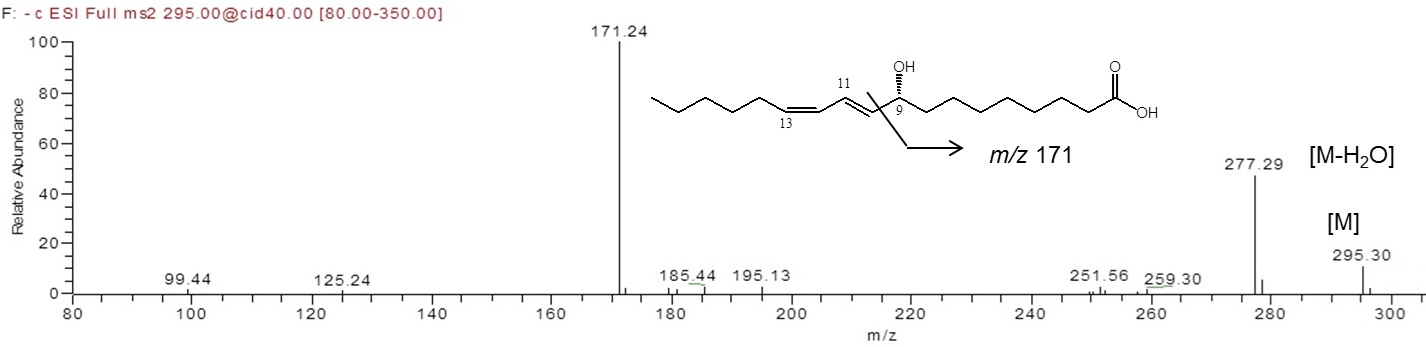

Supplement: S1 Fig — The reaction was performed in 50 mM Tris-HCl buffer (pH 8.5), 10 mM LA, and 0.25 U ml−1 enzyme at 25°C for 5 min. (TIF) [file pone.0137785.s001.tif]

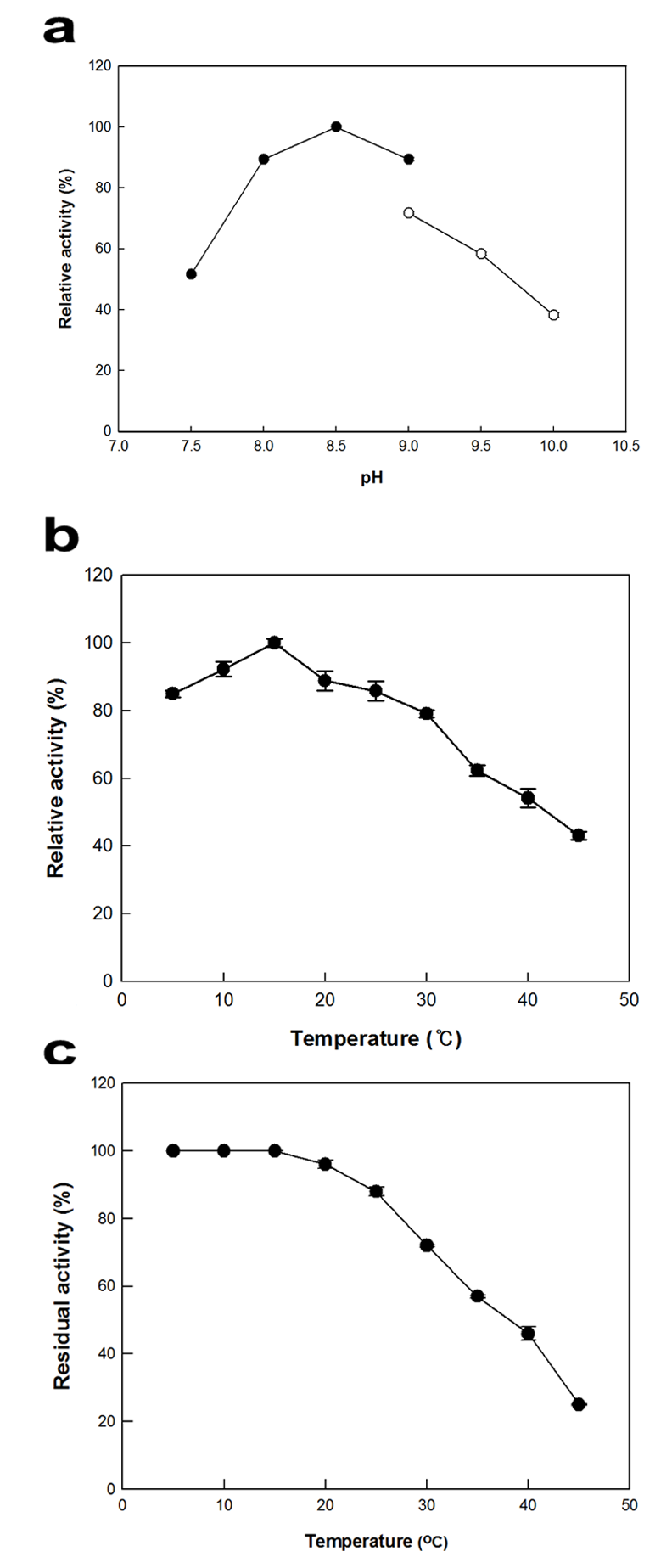

Supplement: S2 Fig — The data represent the means of three separate experiments, and error bars represent the standard deviation. a Effect of pH. The reactions were performed by varying the pH from 7.0 to 10.0 using 50 mM Tris-HCl buffer (pH 7.0–9.0) (filled circle) and 50 mM sodium borate buffer (pH 9.0–10.0) (empty circle) containing 0.25 mM ALA and 0.05 U/mL enzyme at a constant temperature of 25°C for 5 min. b Effect of temperature. The reactions were performed by varying the temperature from 10 to 50°C in 50 mM Tris-HCl buffer (pH 8.5) containing 0.25 mM ALA and 0.05 U ml−1 enzyme for 5 min. c The effect of temperature on 9R-LOX stability. Experiments were carried out after incubation at temperatures ranging from 4 to 45°C for 4 h. Samples were withdrawn at time intervals and then assayed in 50 mM Tris-HCl buffer (pH 8.5) containing 0.25 mM ALA and 0.05 U ml−1 enzyme at 15°C for 5 min. (TIF) [file pone.0137785.s002.tif]

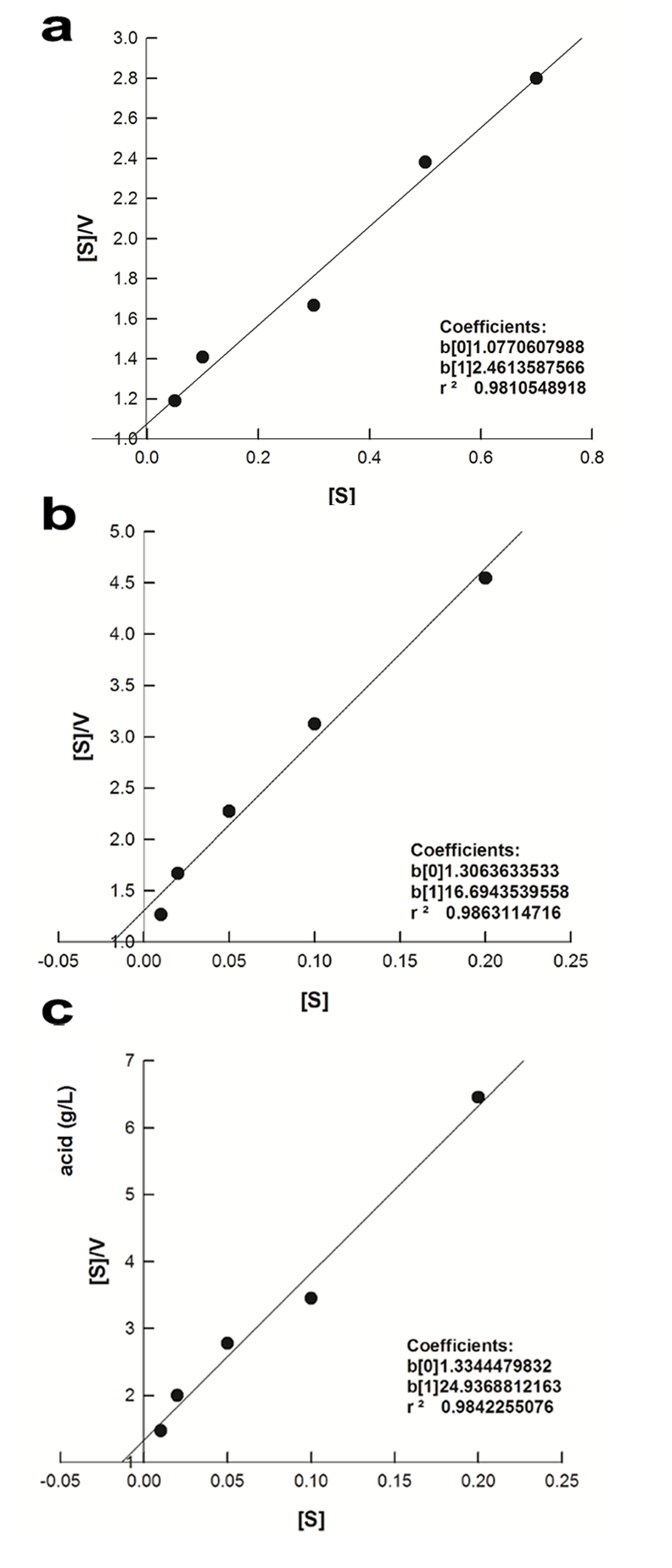

Supplement: S3 Fig — a Reacted with LA. b Reacted with ALA. c Reacted with GLA. (TIF) [file pone.0137785.s003.tif]

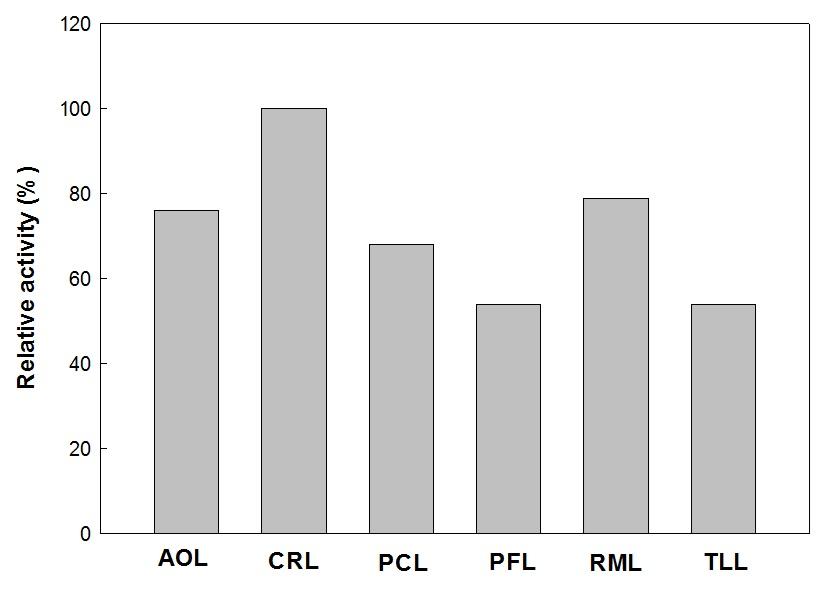

Supplement: S4 Fig — The reactions were performed in 50 mM Tris-HCl buffer (pH 7.5), 1 U ml−1 lipase, and 0.1 g−1 L−1 of PO at 30°C for 30 min. Recombinant AOL, CRL, TLL, RML, PCL, and PFL were used for the hydrolysis of PO. (TIF) [file pone.0137785.s004.tif]
