# Supplementary material for: Selective Production of 9R-Hydroxy-10E,12Z,15Z-Octadecatrienoic Acid from α-Linolenic Acid in Perilla Seed Oil Hydrolyzate by a Lipoxygenase from Nostoc Sp. SAG 25.82
Source: PLoS One. 2015 Sep 17;10(9):e0137785. doi: 10.1371/journal.pone.0137785 (PMC4574779; doi:10.1371/journal.pone.0137785)
Supplement: S1 Table — (DOCX) [file pone.0137785.s005.docx]

| **Fatty acid** | **Composition (w/w, %)** |
| --- | --- |
| C16:0 | 6 ± 0.2 |
| C18:0 | 2 ± 0.1 |
| C18:1, n-7 | 17 ± 0.6 |
| C18:2, n-6 (LA) | 15 ± 0.3 |
| C18:3, n-3 (ALA) | 60 ± 4 |
| C18:3, n-6 (GLA) | ND |
| Total | 100 |
| SFA | 8 ± 0.2 |
| MUFA | 17 ± 0.6 |
| PUFA | 75 ± 3 |
| Total | 100 |

ND: not detected, SFA: Saturated fatty acid, MUFA: Monounsaturated fatty acid, PUFA: Polyunsaturated fatty acid.

The data represent the means of three separate experiments, and ± represents the standard deviation.
